# Technological Advancements in Male Infertility Microsurgery

**DOI:** 10.3390/jcm10184259

**Published:** 2021-09-20

**Authors:** Nahid Punjani, Caroline Kang, Richard K. Lee, Marc Goldstein, Philip S. Li

**Affiliations:** 1 Center for Male Reproductive Medicine and Microsurgery, Cornell Institute for Reproductive Medicine, Weill Cornell Medicine, Cornell University, New York, NY 10065, USA; nap4001@med.cornell.edu (N.P.); cak4005@med.cornell.edu (C.K.); mgoldst@med.cornell.edu (M.G.); 2 Department of Urology, Weill Cornell Medical College, Cornell University, New York, NY 10065, USA; ril9010@med.cornell.edu

**Keywords:** male infertility, microsurgery, operating microscope, artificial intelligence

## Abstract

There have been significant advancements in male infertility microsurgery over time, and there continues to be significant promise for new and emerging techniques, technologies, and methodologies. In this review, we discuss the history of male infertility and the evolution of microsurgery, the essential role of education and training in male infertility microsurgery, and new technologies in this space. We also review the potentially important role of artificial intelligence (AI) in male infertility and microsurgery.

## 1. Introduction

Of the 12% of couples worldwide with infertility, a male factor is involved in up to 50% of cases [1]. Historically, there were limited treatment options for men with severe male factor infertility, azoospermia, or no sperm in the ejaculate. However, there have been significant advancements to male infertility surgical methods, including the use of microsurgery, which has permitted more options and improved outcomes in these men [2]. That being said, there remains significant room for advancement in male infertility microsurgical methods and technology.

## 2. History of Male Infertility Microsurgery

Prior to the standard operating microscope that we know today, operating spectacles were first used in the 1860s as corrective lenses, which were eventually modified for optical magnification. Carl Zeiss opened a microscope workshop in Germany in 1848, and Zeiss became the first mass producer of high-quality microscopes in the mid-to-late 1800s. Microscopes were first introduced into the operating room in otolaryngology at the University of Sweden in 1921, where Dr. Carl Olof Nylen modified a dissecting microscope to perform middle ear procedures [3,4,5,6]. Following this, Dr. Richard Peritt began using an “operating” microscope in ophthalmology in 1946 at Loyola University for cataract surgery [3]. The concept of an operating microscope was introduced into urology in the 1970s, and it was first used to treat vasal epididymal obstruction by Drs. Owen and Silber [3,7,8]. From here, microsurgery has greatly expanded, and has become the standard in reproductive microsurgeries in urology for many procedures, including vasal reconstruction, varicocele repair, and sperm retrieval. It has resulted in significant improvements in clinical outcomes, as well as the minimization of complications and unwanted iatrogenic injuries.

### 2.1. Vasal Obstruction

Vasal obstruction most commonly occurs secondary to prior vasectomy, a procedure where the vas deferens is intentionally severed as a method of permanent contraception. The process of reconnecting the two cut ends of the vas deferens, or “vasovasostomy (VV)”, is a technically and mentally challenging anastomosis, as the human vasal lumen is approximately 250 um (0.25 mm) in diameter [3]. Furthermore, if intraoperative testing determines an epididymal obstruction is present, a connection between the vasal lumen and an epididymal tubule, termed “vaso-epididymostomy (VE)”, the most technically challenging microsurgical anastomosis, must be performed. Initially, patency rates were low because these procedures, which were performed by urologists at that time, resulted in fistula formation between the vas deferens and the epididymis [3]. Dr. Silber reported the first end-to-end microscopic VE in 1978, and, in the 1980s, Dr. Fogdestam described an anastomosis of the vasal cut end to the side of an epididymal tubule [3,9,10,11]. In 1999, a triangulation end-to-side intussusception VE was introduced by Dr. Berger, and it was modified by Dr. Chan et al., as a longitudinal two suture VE in 2003 [12,13]. In this procedure, an epididymal tubule was sutured to the cut end of the vas deferens, but anastomosed in such a way as to invaginate the epididymal tubule into the vasal lumen [3,12,14,15]. These technical advances, including a single-armed longitudinal VE that was developed at Weill Cornell in 2007, have greatly improved patency success rates following VV and VE [16].

### 2.2. Varicoceles

Varicoceles were first described as a pathological process in the first century AD [17] There have been numerous approaches to repairing varicoceles, including laparoscopic, open, or embolization approaches at various access sites to the spermatic cord (i.e., retroperitoneal, inguinal, subinguinal) [17]. In the 1990s, Dr. Marmar and Dr. Goldstein began the practice of performing varicocele repair at the subinguinal level microscopically, to help delineate the spermatic veins from the gonadal arteries [3,18,19,20] At present, microscopic subinguinal varicocelectomy is considered the gold standard in varicocele repair, given that evidence from a meta-analysis and systematic review indicates the microsurgical method as the least morbid and with the highest success rate [21].

### 2.3. Microsurgical Sperm Retrieval

Testicular sperm extraction was first developed only after advancements in the field of reproductive medicine were made. In the 1970s, ejaculated sperm from a fertile man was used for in vitro fertilization (IVF) and resulted in a live birth [22]. Following this, in the 1980s, sperm was surgically retrieved from a man with obstructive azoospermia for use in IVF [23]. Intracytoplasmic sperm injection (ICSI) was introduced in 1992, requiring only a single spermatozoon to fertilize an oocyte [24]. In 1995, testicular sperm extraction was performed on a man with non-obstructive azoospermia (NOA) and successfully used in IVF-ICSI [25]. Testicular sperm extraction was conventionally performed by obtaining testicular tissue through aspiration (testicular sperm aspiration, TESA) or open biopsy (testicular sperm extraction, TESE). However, in 1998, Drs. Schlegel and Li presented a video of microdissection testicular sperm extraction (mTESE), a technique where the testicle is bivalved, and the size and color of individual tubules are analyzed for the most likely tubules to contain sperm [26,27]. While there have been reports, as well as a large systematic review, and meta-analysis suggesting similar sperm retrieval rates between conventional and mTESE, at least in the NOA population, these data should be interpreted cautiously as it is mainly based on non-randomized and heterogeneous data [2,28]. The one randomized study in this review did suggest a benefit for mTESE [2]. Importantly, this technique is notably less traumatic to the blood supply of the testis as the vasculature can be directly visualized, and sperm retrieval rates are relatively high, given the ability to directly visualize the seminiferous tubules prior to harvest [26,29].

## 3. Training and Male Infertility Microsurgery

Male infertility microsurgical procedures comprise some of the most technically challenging surgical operations, and thus, appropriate training under the operating microscope is imperative to optimal outcomes [30,31]. Currently, the majority (approximately 80%) of Accreditation Council for Graduate Medical Education accredited residencies have a fellowship-trained microsurgeon on faculty, allowing young urological surgeons early exposure to microsurgery [32]. However, performing a procedure only several times may not provide the expertise that one needs to be successful with male infertility microsurgical procedures. The ability to view and identify critical tissues under the appropriate magnification, and perform delicate anastomoses, is critical to the success of the procedure, and to minimize adverse outcomes for patients. Objective evaluation of skills acquired during microsurgical training is vital in documenting progress and allowing for constructive feedback on areas to improve [31]. Having a microsurgical laboratory with a dedicated laboratory director experienced in microsurgery is critical to the success of the trainees within that laboratory [30,31]. Learning the appropriate hand placement, hand movements, suture placement, and knot-tying in a safe environment are crucial to eventual success within the operating room [30,31]. Additional methods for training and practice outside the operating room include operating microscope simulation devices which can attach to smartphones as well as training in smartphone applications.

Ultimately, proficiency in male infertility microsurgical procedures should be learned in a microsurgical training lab, not directly on patients. Success in male infertility microsurgery is heavily dependent on the quality of practice and training in the microsurgical lab. A surgeon’s hand–eye coordination, dexterity, and steadiness can be developed and optimized during practice in the lab with adequate supervision and instruction. This is incredibly important as the clinical outcomes for male infertility microsurgery are not always evident at the end of the surgical procedure. 

## 4. Video Microsurgery and 4K3D Operating Microscopes

Since the development of the operating microscope and introduction into urology, the equipment has become more sophisticated, quality of the magnification has improved, and it has become more ergonomic and more aesthetic. This allows for the incorporation of high definition displays, and has permitted multiple attachments for assistants [33]. However, the fundamental principles of two-dimensional standard operating microscopes are limited to varying levels of magnification and limited short working distance. With the recent significant technological improvement within the video and digital industry, 4K3D video operating microscopes have become a real potential game-changer for male infertility and microsurgery [34,35,36]. This technology can provide comparable, if not superior, magnification, uses large flat screens, which allows visualization and better ergonomics for all members of the operating team, and has less bulky equipment [37]. Recent advancements in development of video microscopes utilize not only three-dimensional technology, which may improve operative visualization and depth perception, but also provide enhanced 4K resolution in real-time [38]. The 4K3D microscope also possesses a digital zoom system which assists surgeons in discriminating anatomical detail and improves surgeon posture with a much more ergonomic operating position. Additionally, the technology offers additional benefits of a larger and broader color range that may facilitate finer tissue discrimination during microsurgery. Finally, this plug-and-play, easy-to-transport technology permits not only real-time surgical visualization but also video sharing capabilities, which provides an excellent platform for surgical training. 

**Figure 1:**
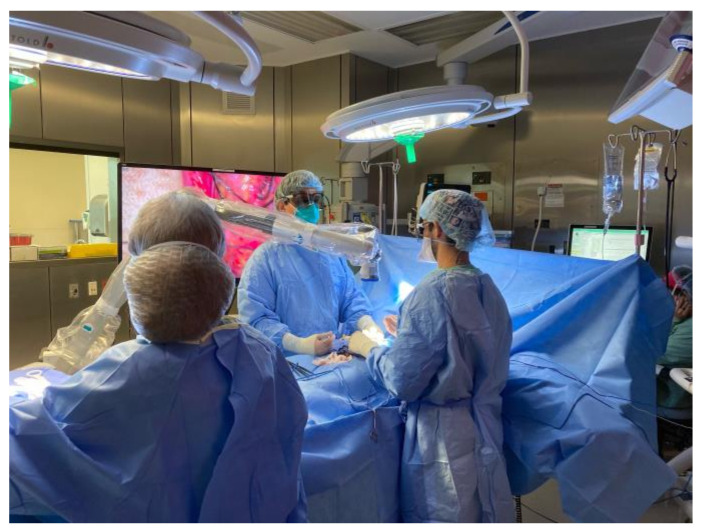
Olympus 4K3D Orbeye video microscope being utilized for male infertility microsurgery. Both surgeons are able to view large, high-definition screens with a less bulky microscope. (Reprinted with permission from [34]).

Studies in male microsurgery have demonstrated that these technologies have promising improvements in operating times, and turnover time between cases, as well as an improvement in surgeon ergonomics. They also have non-inferior outcomes in animal models [34,39]. The set-up, as seen in Figure 1, requires less operating room space, and its design also facilitates easy transportability. This may also facilitate opportunities for trainee practice outside of the operating room, and supervision by multiple individuals, given the large high-definition screens. Furthermore, its promise has been shown to varying degrees in numerous other subspecialties, including plastic surgery, neurosurgery, general surgery, and vascular surgery [40,41,42]. However, given the enormous upfront costs associated with purchasing a new device, and the lack of comfort for surgeons, the uptake continues to be slow.

## 5. Robotics and Male Infertility Microsurgery

Robotic surgery was first utilized in the field of orthopedic and neurosurgery in the 1980s, with the intention of three dimensional visualization, surgical reproducibility, precision, and an ability to perform surgery from a distance [43]. Robotics have since had uptake in many other specialties, with its first use in urology in 1989, and has now become the standard method for many procedures [43]. The daVinci^®^ surgical robot is currently the only USA Food and Drug Administration-approved device, and within male infertility, it has evolved as a possible adaptation from traditional microsurgery, given its potential benefits such as improved visualization, ergonomics, reduction in tremor, and potential to obviate the need for a surgical assistant [44,45]. 

The first prospective randomized study of robotic male infertility microsurgery was a study in an animal model reported by Drs. Schiff, Li, and Goldstein in 2004 [46]. Since this time, robotic microsurgery has been completed for vasectomy reversal in humans. While there have been multiple published reports, to date, there continues to be limited widespread adoption [44]. One such study demonstrated comparable clinical outcomes, but only demonstrated reduced operative time after a higher number of cases were completed [47]. Other procedures which have been examined include varicocelectomy, spermatic cord denervation, and microdissection testicular sperm extraction, but again the uptake has been limited [44]. However, an additional benefit of robotics in male infertility may be for complex cases which may require a large incision for an open procedure. This may require deep access to areas such as the pelvis, which may allow for much smaller port incisions [47]. To date, as previously discussed, reasons for ongoing slow adoption include limited high-quality data, limited feedback on delicate tissue handling ability, cost, and lack of benefit over other technologies such as video microsurgery [44]. A recent expert debate also suggests that at this time, no substantial clinical evidence exists suggesting improved outcomes, but instead may require extra microsurgical training coupled with extensive costs that come with robotic surgery [48].

## 6. Multiphoton Microscopy

With variable success rates of sperm retrieval due to differing etiologies for non-obstructive azoospermia, and the non-insignificant impacts on testis tissue following biopsy or extensive microdissection, additional technologies have been explored to assist in sperm retrieval during mTESE. One such technology is multiphoton microscopy, which was first used in the 1990s, and was suggested as an adjunctive technology tool for enhanced visualization during surgical sperm retrieval [49,50]. Multiphoton microscopy utilizes nonlinear excitation for fluorescence and is commonly used for biological imaging, as it provides real-time imaging [50]. 

**Figure 2:**
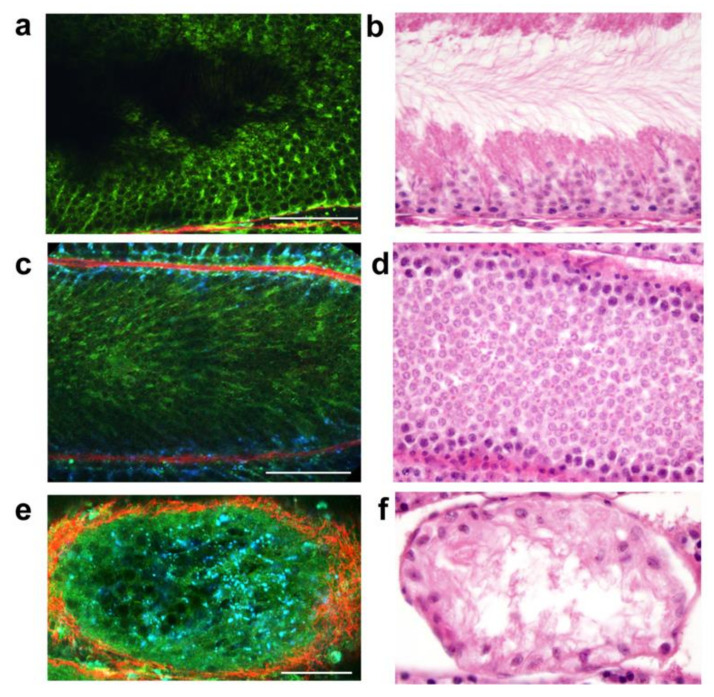
Varying seminiferous tubule histology patterns from rat testis procured using an ischemia hypothermia model (multiphoton microscopy panels (**a**,**c**,**e**); fixed in Bouin’s solution panels (**b**,**d**,**f**). Panels (**a**,**b**) show seminiferous tubules with spermatogenesis. Panels (**c**,**d**) demonstrate tubules with maturation arrest. Panels (**e**,**f**) illustrate tubules with a Sertoli cell only pattern. (Reprinted with permission from [49]. Copyright 2011 American Urological Association).

While it has been used in other areas of urology such as treatment of urologic malignancies, multiphoton microscopy use has been limited in the management of male infertility. One animal study was performed which demonstrated the ability to identify specific stages of real-time spermatogenesis within a seminiferous tubule, which is theorized to be secondary to a difference in steroid metabolism in Sertoli cells at these different stages (Figure 2) [49]. It is important to note that there was originally some concern regarding increased sperm DNA damage from the laser utilized during multiphoton microscopy, but this animal study demonstrated minimal DNA damage [51]. Use in humans is limited, with only a single study conducted that examined tissue at the time of testicular biopsy, and it demonstrated similar findings, that may generally be identified with hematoxylin and eosin staining [52]. Unfortunately, despite the potential promise of multiphoton microscopy, its use has been limited, due to further required work in human models, optimization of safety, and ongoing evaluation of its possible use in non-operative room settings [50]. In addition to multiphoton microscopy, other methods have also been considered as adjunctive technologies, such as narrow-band imaging, which permits blood vessel visualization to determine active areas of spermatogenesis [53].

## 7. Artificial Intelligence, Deep Learning and Machine Learning

Machine learning (ML) is a concept that falls under the broad category of AI, a technique that uses machines to mimic human intelligence. Deep learning (DL) is a further subset of ML which uses neural networks. ML specifically uses statistical techniques and algorithms to learn without explicit programming and incorporates various features (i.e., variables or attributes) to complete or compute specific tasks [54]. These concepts were first introduced in the 1950s by Alan Turing, who suggested the ability of a machine to replicate human capabilities [55]. Since this time, both AI and DL/ML have made strong inroads into the biomedical world and are now used in a wide variety of applications [56]. Common uses of AI in healthcare include examination of images such as radiographic images and pathologic specimens, both of which are some of the ways AI is utilized in the field of urology [56]. These concepts have their most significant potential benefit where there exist large sets of data to analyze, and numerous variables to develop the best and most accurate algorithms. 

ML may be classified as supervised, that is, algorithm development with knowledge of the outcome of interest, unsupervised, which aims to look for the relationship between various features without a known outcome, or a mix of supervised and unsupervised, also known as semi-supervised learning [57]. The choice of which type of strategy to use depends on the available data, and whether the outcome of interest is known. However, despite the theoretical power and appeal of ML, its usability falls on a spectrum of interpretability and accuracy, which is the ultimate ML tradeoff [58]. For example, techniques such as neural networks are highly accurate, yet can be challenging to interpret, whereas regression techniques and decision trees are much more interpretable but come with less accuracy.

### 7.1. AI and Microsurgery

The use of AI and ML has been studied in urology within the realm of urologic oncology, but also in other benign subspecialties, including endourology and pediatric urology [56,59]. However, one area that AI/ML may be applicable to all realms of urology is surgical training, particularly in robotic and microsurgical training. Previous studies have examined the use of ML/AI algorithms in robotic surgery to assess, analyze, and evaluate surgical skills and performance, and subsequently utilize this information to predict surgical outcomes [56,59]. AI takes advantage of the large amount of data that may be obtained from the visual screen on an operating robot, and, therefore, it is plausible that this could be applied to microsurgery. With the increasing use of video microscopes, AI/ML may be applied, and is an area that requires further study. 

### 7.2. AI and Infertile Men

As previously described, since AI/ML benefits from large sets of data, there is a logical and potential natural role in male infertility [60]. Male infertility is heavily rooted in a thorough history and physical exam, given the limited ancillary tests, which include a semen analysis, serum hormones and at times additional specialized ancillary tests. AI/ML algorithms may theoretically incorporate this data, to help develop an algorithm to aid in infertility diagnosis. One such example of this application includes a published report which utilized artificial neural networks to predict patients with azoospermia who should undergo genetic evaluation [61]. 

With growing evidence of the role of systemic and possible genetic disease in men with male infertility, there has been greater emphasis on more advanced genetic screening, such as whole-exome sequencing [62]. This technique garners large amounts of data, and, therefore, ML algorithms and methods have a pivotal role to play in genetics and genomics [63].

### 7.3. AI and Semen Analysis/Sperm Selection

AI was first used in the field of male infertility in the analysis of semen quality. Several reports in the early 2010s examined associations between environmental and lifestyle factors and their impact on semen quality and others using general questionnaire data. Many of these studies used artificial neural networks and a ML algorithm, for analyzing the data [64,65]. Since this time, other studies have explored various AI/ML algorithms incorporating different features, all with the ultimate goal of predicting sperm quality [66,67]. One study also aimed to predict, using both clinical and demographic variables, the presence or absence of sperm in non-obstructive azoospermia biopsy specimens with reasonable accuracy [68].

AI has also been used in studies of sperm selection for use in assisted reproductive technologies (ART). This is important because ART (including in-vitro fertilization and intracytoplasmic sperm injection), require optimal sperm selection for subsequent oocyte injection. While methods exist to aid in sperm selection, such as the swim-up or density- gradient centrifugation methods, there still remains significant subjectivity in sperm selection, which AI/ML may help optimize and make more objective [69,70]. Attempts have been made to automate semen analysis with computer-assisted semen analysis, but despite improved objectivity, it has been faced with decreased reproducibility, and, therefore, has not been adopted in many clinics for routine use [70,71]. There also are newer sperm selection technologies, such as microfluidic cell sorting, a technique that uses small fluid streams to sort cells, and magnetic-activated cell sorting and fluorescence-activated cell sorting, which may revolutionize sperm selection when coupled with AI/ML algorithms [70,72,73]. 

In addition to sperm selection, AI methods have also been studied in oocyte, gamete, and embryo selection [71,74]. Studies are, however, limited to date for real-time intraoperative sperm selection. That said, AI methods have the potential to be leveraged for real-time application [73]. These advancements could assist microsurgeons in the operating room, as well as with extensive sperm search in the lab. Real-time adaptations could prioritize “top spots,” that is areas with particular visual characteristics, which would contain seminiferous tubules that may be more likely to possess sperm during surgical sperm retrieval. This may result in potentially superior outcomes, and more accurate microsurgical dissection.

### 7.4. Limitations of AI/ML in Male Infertility

Unfortunately, to date, many AI studies have originated from single centers and are based on smaller cohorts [73]. Furthermore, there are well-known challenges in the reproducibility of these algorithms, and, therefore, they may negatively impact the generalizability of AI/ML. Future work is warranted in AI/ML and reproductive urology.

## 8. Limitations and Future Perspectives

Despite the creation of new technology (Table 1), as well as new potential opportunities, these technologies often come at a significant cost. This includes sizeable and significant financial purchasing costs, maintenance, storage, quality control, and substantial time and effort spent in learning and adaptation. This must all be considered, as each new technology does not necessarily reduce operative time or improve functional outcomes. Therefore, while many of these technologies continue to be promising, these factors must not be underrated.

## 9. Conclusions

The future of male infertility and microsurgery is bright. Since the discovery and adaptation of microsurgery in male infertility, significant advancements have been made, and have gradually become the standard for multiple procedures. In addition, there are many potentially game-changing technologies and concepts, including video microscopy, robotic surgery, adjunctive imaging technologies, and artificial intelligence, which may revolutionize the field of male infertility. 

## Figures and Tables

**Table 1:** Pros and cons of technologies in male infertility microsurgery.

Technology	Pros	Cons
Video Microsurgery and 4K3D Operating Microscopes [34,35,36]	More ergonomic High definition/quality displays Easy transport Less space	Expensive upfront cost Learning curve Surgeon comfort
Robotics and Male Infertility Microsurgery [44,46,48]	Reduce tremor Additional arm can replace an assistant Improved visualization	Large upfront cost Requires extra microsurgical robotic and male infertility microsurgery training No concrete clinical evidence suggesting better outcomes Extra microsurgical training required Large space and operating room staff
Multiphoton Microscopy [49,50,52,75,76]	Identification of real-time spermatogenesis Potentially reduce unnecessary dissection	Safety concerns Technological limitations Cost and learning curve Limited human studies
Artificial Intelligence, Deep Learning and Machine Learning [60,64,65,74]	Powerful Efficient Novel	Interpretability can be challenging May require significant computational power Requires further research

## Data Availability

Not applicable.

## References

[1] Kumar N., Singh A.K. (2015). Trends of male factor infertility, an important cause of infertility: A review of literature. J. Hum. Reprod. Sci..

[2] Punjani N., Kang C., Schlegel P. (2021). Two Decades from the Introduction of Microdissection Testicular Sperm Extraction: How This Surgical Technique Has Improved the Management of NOA. J. Clin. Med..

[3] Chen M.L., Buncke G.M., Turek P.J. (2021). Narrative review of the history of microsurgery in urological practice. Transl. Androl. Urol..

[4] Dohlman G.F. (1969). Carl Olof Nylen and the Birth of the Otomicroscope and Microsurgery. Arch. Otolaryngol. Head Neck Surg..

[5] Schultheiss D., Denil J. (2002). History of the microscope and development of microsurgery: A revolution for reproductive tract surgery. Andrologia.

[6] Kriss T.C., Kriss V.M. (1998). History of the Operating Microscope: From Magnifying Glass to Microneurosurgery. Neurosurgery.

[7] Owen E.R. (1977). Microsurgical Vasovasostomy: A Reliable Vasectomy Reversal. ANZ J. Surg..

[8] Silber S.J. (1977). Microscopic vasectomy reversal. Fertil Steril.

[9] Silber S.J. (1978). Microscopic vasoepididymostomy: Specific microanastomosis to the epididymal tubule. Fertil Steril.

[10] Thomas A.J. (1987). Vasoepididymostomy. Urol. Clin. N. Am..

[11] Fogdestam I., Fall M., Nilsson S. (1986). Microsurgical epididymovasostomy in the treatment of occlusive azoospermia. Fertil Steril.

[12] Chan P.T., Li P.S., Goldstein M. (2003). Microsurgical Vasoepididymostomy: A Prospective Randomized Study of 3 Intussusception Techniques in Rats. J. Urol..

[13] Berger R.E. (1998). Triangulation end-to-side vasoepididymostomy. J. Urol..

[14] Shekarriz M., Pomer S. (1991). Microsurgical vasoepididymostomy: A comparison between the end-to-side anastomosis and the in-vagination technique. Urol. Res..

[15] Stefanović K.B., Clark S.A., Buncke H.J. (1991). Microsurgical Epididymovasostomy by Loop Intussusception. BJU Int..

[16] Monoski M.A., Schiff J., Li P.S., Chan P.T., Goldstein M. (2007). Innovative single-armed suture technique for microsurgical vaso-epididymostomy. Urology.

[17] Kang C., Punjani N., Lee R.K., Li P.S., Goldstein M. (2021). Effect of varicoceles on spermatogenesis. Semin. Cell Dev. Biol..

[18] Marmar J., Kim Y. (1994). Subinguinal Microsurgical Varicocelectomy: A Technical Critique and Statistical Analysis of Semen and Pregnancy Data. J. Urol..

[19] Gorelick J.I., Goldstein M. (1993). Loss of fertility in men with varicocele. Fertil Steril.

[20] Steckel J., Dicker A., Goldstein M. (1993). Relationship Between Varicocele Size and Response to Varicocelectomy. J. Urol..

[21] Ding H., Tian J., Du W., Zhang L., Wang H., Wang Z. (2012). Open non-microsurgical, laparoscopic or open microsurgical vari-cocelectomy for male infertility: A meta-analysis of randomized controlled trials. BJU Int..

[22] Wang J., Sauer M.V. (2006). In vitro fertilization (IVF): A review of 3 decades of clinical innovation and technological advancement. Ther. Clin. Risk Manag..

[23] Temple-Smith P.D., Southwick G.J., Yates C.A., Trounson A., De Kretser D.M. (1985). Human pregnancy by in vitro fertilization (IVF) using sperm aspirated from the epididymis. J. Assist. Reprod. Genet..

[24] Palermo G., Joris H., Devroey P., Van Steirteghem A.C. (1992). Pregnancies after intracytoplasmic injection of single spermatozoon into an oocyte. Lancet.

[25] Devroey P., Liu J., Nagy Z., Goossens A., Tournaye H., Camus M., van Steirteghem A., Silber S. (1995). Pregnancies after testicular sperm extraction and intra-cytoplasmic sperm injection in non-obstructive azoospermia. Hum. Reprod..

[26] Schlegel P.N. (1999). Testicular sperm extraction: Microdissection improves sperm yield with minimal tissue excision. Hum. Reprod..

[27] Schlegel P.N., Li P.S. (1998). Microdissection TESE: Sperm retrieval in non-obstructive azoospermia. Hum. Reprod. Update.

[28] Corona G., Minhas S., Giwercman A., Bettocchi C., Dinkelman-Smit M., Dohle G., Fusco F., Kadioglou A., Kliesch S., Kopa Z. (2019). Sperm recovery and ICSI outcomes in men with non-obstructive azoospermia: A systematic review and meta-analysis. Hum. Reprod. Updat..

[29] Ramasamy R., Yagan N., Schlegel P.N. (2005). Structural and functional changes to the testis after conventional versus microdissection testicular sperm extraction. Urology.

[30] Mehta A., Li P.S. (2013). Male infertility microsurgical training. Asian J. Androl..

[31] Mehta A., Li P.S., Goldstein M. (2014). Male infertility microsurgical training. Transl. Androl. Urol..

[32] Masterson T.A., Nackeeran S., Rainer Q., Hauser N., Marcovich R., Ramasamy R. (2021). Survey of Microsurgery Training Availability in US Urology Residency Programs. World J. Men’s Health.

[33] Uluç K., Kujoth G., Başkaya M.K. (2009). Operating microscopes: Past, present, and future. Neurosurg. Focus.

[34] Best J.C., Gonzalez D., Alawamlh O.A.H., Li P.S., Ramasamy R. (2020). Use of 4K3D video microscope in male infertility microsurgery. Urol. Video J..

[35] Hayden R.P., Chen H., Li P.S., Goldstein M. (2019). Promising 4K3D Reconstructive Macrosurgery and Microsurgery. AUA News.

[36] Chen H., Hayden R.P., Al Hussein Alawamlh O., Schlegel P.N., Goldstein M., Li P.S. (2020). New era of male infertility microsurgery: 4K3D ORBEYE video operating microscopy. Fertil Steril.

[37] Frykman P.K., Duel B.P., Gangi A., Williams J.A., Berci G., Freedman A.L. (2013). Evaluation of a Video Telescopic Operating Microscope (VITOM) for Pediatric Surgery and Urology: A Preliminary Report. J. Laparoendosc. Adv. Surg. Tech..

[38] Wahba R., Datta R., Bußhoff J., Bruns T., Hedergott A., Gietzelt C., Dieplinger G., Fuchs H., Morgenstern B., Möller D. (2020). 3D Versus 4K Display System–Influence of “State-of-the-art”—Display Technique on Surgical Performance (IDOSP-study) in Minimally Invasive Surgery: A Randomized Cross-over Trial. Ann. Surg..

[39] Hayden R.P., Chen H., Goldstein M., Li P.S. (2019). A randomized controlled animal trial: Efficacy of a 4K3D video microscope versus an optical operating microscope for urologic microsurgery. Fertil Steril.

[40] Ahmad F.I., Mericli A.F., DeFazio M.V., Chang E.I., Hanasono M.M., Pederson W.C., Kaufman M., Selber J.C. (2019). Application of the ORBEYE three-dimensional exoscope for microsurgical procedures. Microsurgery.

[41] Takahashi S., Toda M., Nishimoto M., Ishihara E., Miwa T., Akiyama T., Takashi H., Hikaru S., Kazunari Y. (2018). Pros and cons of using ORBEYE for microneuro-surgery. Clin. Neurol. Neurosurg..

[42] Izumo T., Ujifuku K., Baba S., Morofuji Y., Horie N., Matsuo T. (2019). Initial experience of ORBEYE™ surgical microscope for carotid endarterectomy. Asian J. Neurosurg..

[43] Challacombe B., Khan M.S., Murphy D., Dasgupta P. (2006). The history of robotics in urology. World J. Urol..

[44] Darves-Bornoz A., Panken E., Brannigan R.E., Halpern J.A. (2020). Robotic Surgery for Male Infertility. Urol. Clin. N. Am..

[45] Pastuszak A.W., Wenker E.P., Lipshultz L.I. (2015). The History of Microsurgery in Urology. Urology.

[46] Schiff J., Li P.S., Goldstein M. (2004). Robotic microsurgical vasovasostomy and vasoepididymostomy: A prospective randomized study in a rat model. J. Urol..

[47] Etafy M., Gudeloglu A., Brahmbhatt J.V., Parekattil S.J. (2017). Review of the role of robotic surgery in male infertility. Arab. J. Urol..

[48] Chan P., Parekattil S.J., Goldstein M., Lipshultz L.I., Kavoussi P., McCullough A., Sigman M. (2018). Pros and cons of robotic microsurgery as an appropriate approach to male reproductive surgery for vasectomy reversal and varicocele repair. Fertil Steril.

[49] Ramasamy R., Sterling J., Fisher E.S., Li P.S., Jain M., Robinson B.D., Maria S., David H., Chris X., Sushmita M. (2011). Identification of spermatogenesis with multiphoton mi-croscopy: An evaluation in a rodent model. J. Urol..

[50] Katz M.J., Huland D.M., Ramasamy R. (2014). Multiphoton microscopy: Applications in Urology and Andrology. Transl. Androl. Urol..

[51] Chiba K., Enatsu N., Fujisawa M. (2016). Management of non-obstructive azoospermia. Reprod. Med. Biol..

[52] Najari B.B., Ramasamy R., Sterling J., Aggarwal A., Sheth S., Li P.S., Dubin J.M., Goldenberg S., Jain M., Robinson B.D. (2012). Pilot Study of the Correlation of Multiphoton Tomography of Ex Vivo Human Testis with Histology. J. Urol..

[53] Enatsu N., Miyake H., Chiba K., Fujisawa M. (2015). Identification of Spermatogenically Active Regions in Rat Testes by Using Nar-row-band Imaging System. Urology.

[54] Bi Q., Goodman K.E., Kaminsky J., Lessler J. (2019). What is Machine Learning?. A Primer for the Epidemiologist. Am. J. Epidemiol..

[55] French R.M. (2000). The Turing Test: The first 50 years. Trends Cogn. Sci..

[56] Chang T.C., Seufert C., Eminaga O., Shkolyar E., Hu J.C., Liao J. (2021). Current Trends in Artificial Intelligence Application for En-dourology and Robotic Surgery. Urol. Clin. N. Am..

[57] Sidey-Gibbons J.A.M., Sidey-Gibbons C.J. (2019). Machine learning in medicine: A practical introduction. BMC Med Res. Methodol..

[58] Murdoch W.J., Singh C., Kumbier K., Abbasi-Asl R., Yu B. (2019). Definitions, methods, and applications in interpretable machine learning. Proc. Natl. Acad. Sci. USA.

[59] Hameed B., Dhavileswarapu A.S., Raza S., Karimi H., Khanuja H., Shetty D., Ibrahim S., Shah M., Naik N., Paul R. (2021). Artificial Intelligence and Its Impact on Urological Diseases and Management: A Comprehensive Review of the Literature. J. Clin. Med..

[60] Chu K.Y., Nassau D.E., Arora H., Lokeshwar S.D., Madhusoodanan V., Ramasamy R. (2019). Artificial Intelligence in Reproductive Urology. Curr. Urol. Rep..

[61] Akinsal E.C., Haznedar B., Baydilli N., Kalinli A., Ozturk A., Ekmekçioğlu O. (2018). Artificial Neural Network for the Prediction of Chromosomal Abnormalities in Azoospermic Males. Urol. J..

[62] Punjani N., Lamb D.J. (2020). Canary in the Coal Mine? Male Infertility as a Marker of Overall Health. Annu. Rev. Genet..

[63] Libbrecht M., Noble W.S. (2015). Machine learning applications in genetics and genomics. Nat. Rev. Genet..

[64] Gil D., Girela J.L., De Juan J., Gomez-Torres M.J., Johnsson M. (2012). Predicting seminal quality with artificial intelligence methods. Expert Syst. Appl..

[65] Girela J.L., Gil D., Johnsson M., Gómez-Torres M.J., De Juan J. (2013). Semen Parameters Can Be Predicted from Environmental Factors and Lifestyle Using Artificial Intelligence Methods. Biol. Reprod..

[66] Shi X., Chan C.P.S., Waters T., Chi L., Chan D.Y., Li T.C. (2018). Lifestyle and demographic factors associated with human semen quality and sperm function. Syst. Biol. Reprod. Med..

[67] Jurewicz J., Radwan M., Sobala W., Ligocka D., Radwan P., Bochenek M., Hanke W. (2013). Lifestyle and semen quality: Role of modifiable risk factors. Syst. Biol. Reprod. Med..

[68] Zeadna A., Khateeb N., Rokach L., Lior Y., Har-Vardi I., Harlev A., Huleihel M., Lunenfeld E., Levitas E. (2020). Prediction of sperm extraction in non-obstructive azo-ospermia patients: A machine-learning perspective. Hum. Reprod..

[69] You J.B., McCallum C., Wang Y., Riordon J., Nosrati R., Sinton D. (2021). Machine learning for sperm selection. Nat. Rev. Urol..

[70] Hicks S.A., Andersen J.M., Witczak O., Thambawita V., Halvorsen P., Hammer H.L., Haugen T.B., Riegler M.A. (2019). Machine Learning-Based Analysis of Sperm Videos and Participant Data for Male Fertility Prediction. Sci. Rep..

[71] Wang R., Pan W., Jin L., Li Y., Geng Y., Gao C., Chen G., Wang H., Ma D., Liao S. (2019). Artificial intelligence in reproductive medicine. Reproduction.

[72] Vaughan A.D., Sakkas D. (2019). Sperm selection methods in the 21st century. Biol. Reprod..

[73] Patel D.P., Gross K.X., Hotaling J.M. (2021). Can artificial intelligence drive optimal sperm selection for in vitro fertilization?. Fertil Steril.

[74] Zaninovic N., Rosenwaks Z. (2020). Artificial intelligence in human in vitro fertilization and embryology. Fertil Steril.

[75] Mukherjee S., Ramasamy R., Manzoor M., Jain M., Li P.S., Sterling J., Salamoon B., Fisher E., Schlegel P.N. (2012). Full field optical coherence tomography can identify spermatogenesis in a rodent sertoli-cell only model. J. Pathol. Inform..

[76] Ramasamy R., Sterling J., Li P.S., Robinson B.D., Parekattil S., Chen J., Felsen D., Mukherjee S., Goldstein M., Schlegel P.N. (2012). Multiphoton Imaging and Laser Ablation of Rodent Spermatic Cord Nerves: Potential Treatment for Patients with Chronic Orchialgia. J. Urol..

